# Recombinant Viral Vaccines Expressing Merozoite Surface Protein-1 Induce Antibody- and T Cell-Mediated Multistage Protection against Malaria

**DOI:** 10.1016/j.chom.2008.12.004

**Published:** 2009-01-22

**Authors:** Simon J. Draper, Anna L. Goodman, Sumi Biswas, Emily K. Forbes, Anne C. Moore, Sarah C. Gilbert, Adrian V.S. Hill

**Affiliations:** 1 The Jenner Institute, University of Oxford, Old Road Campus Research Building, Oxford OX3 7DQ, UK

**Keywords:** MICROBIO, CELLIMMUNO

## Abstract

Protecting against both liver and blood stages of infection is a long-sought goal of malaria vaccine design. Recently, we described the use of replication-defective viral vaccine vectors expressing the malaria antigen merozoite surface protein-1 (MSP-1) as an antimalarial vaccine strategy that elicits potent and protective antibody responses against blood-stage parasites. Here, we show that vaccine-induced MSP-1-specific CD4^+^ T cells provide essential help for protective B cell responses, and CD8^+^ T cells mediate significant antiparasitic activity against liver-stage parasites. Enhanced survival is subsequently seen in immunized mice following challenge with sporozoites, which mimics the natural route of infection more closely than when using infected red blood cells. This effect is evident both in the presence and absence of protective antibodies and is associated with decreased parasite burden in the liver followed by enhanced induction of the cytokine IFN-γ in the serum. Multistage immunity against malaria can thus be achieved by using viral vectors recombinant for MSP-1.

## Introduction

Approximately 500 million cases and 1 million deaths occur annually due to *Plasmodium falciparum* malaria infection (Greenwood et al., 2008). The development of an effective vaccine remains an important goal for the safe and cost-effective control of this disease. Blood-stage malaria vaccine development has classically focused on recombinant protein-in-adjuvant formulations (Genton and Reed, 2007); however, we have described the use of replication-defective viral vaccine vectors expressing the malaria antigen merozoite surface protein-1 (MSP-1) as a promising alternative vaccination strategy (Draper et al., 2008).

MSP-1 is a large polypeptide that undergoes proteolytic processing upon erythrocyte invasion, during which the 42 kDa C terminus (MSP-1_42_) is cleaved into 33 kDa (MSP-1_33_) and 19 kDa (MSP-1_19_) fragments (Holder et al., 1987). High-titer antibody responses against MSP-1_19_, but not MSP-1_33_, are reported to account for the protective immunity induced by this antigen (Ahlborg et al., 2002; Yuen et al., 2007). Previously, we have demonstrated that a priming immunization with adenovirus human serotype 5 (AdHu5) followed by a boost with modified vaccinia virus Ankara (MVA), both recombinant for *P. yoelii* MSP-1_42_, can induce potent antibody responses that protect against a lethal challenge with blood-stage parasites.

Viral vaccine vectors, deployed in heterologous prime-boost regimes, have been routinely developed to induce strong T cell responses targeting intracellular pathogens (Hill, 2006), and as expected, the AdHu5-MVA-MSP-1 regime also induced potent MSP-1-specific T cell responses in mice (Draper et al., 2008). However, we demonstrated that depletion of T cell responses in mice prior to blood-stage challenge did not ablate vaccine efficacy, despite the fact that CD4^+^ T cell lines that recognize epitopes within MSP-1_33_ are reported to control the growth of *P. yoelii* following adoptive transfer into immunodeficient mice (Wipasa et al., 2002). Nevertheless, MSP-1 is reported to be expressed within the exoerythrocytic schizonts (Sacci et al., 2005), and T cells against *P. yoelii* MSP-1 have been reported to protect against the liver-stage parasite using a heat shock protein fusion construct (Kawabata et al., 2002). We therefore sought to investigate whether the induction of cellular immune responses, in conjunction with antibody responses, could enhance vaccine efficacy by targeting the parasite at both the liver and blood stages of malaria infection.

Here, we describe in detail the immune mechanisms elicited in mice immunized with AdHu5-MVA-MSP-1 vaccines. We show that CD4^+^ T cell responses against MSP-1_33_ provide essential help for priming B cell responses against MSP-1_19_, while CD8^+^ T cell responses against MSP-1_33_ mediate significant antiparasitic activity against the liver stage. In agreement with protein vaccine studies (Ahlborg et al., 2002), MSP-1_33_-specific responses alone do not mediate blood-stage protection following challenge infection with parasitized red blood cells (pRBCs). However, the AdHu5-MVA-MSP-1 vaccine regimes demonstrate consistently better protection against a lethal blood-stage infection following challenge with *P. yoelii* sporozoites, rather than inoculation with pRBCs. Enhanced efficacy following sporozoite (spz) challenge is associated with vaccine-induced effector mechanisms acting against the liver and blood stages of infection. These data demonstrate that multistage immunity against malaria can be achieved using a single vaccine platform that induces both strong antibody and T cell responses against the MSP-1 antigen.

## Results

### Immunogenicity of AdHu5-MVA-MSP-1 Recombinants

The immunogenicity of heterologous prime-boost immunization using AdHu5 and MVA vectors expressing MSP-1_42_ has been previously reported (Draper et al., 2008), however, we sought to address the contribution of the MSP-1_33_ and MSP-1_19_ component domains of MSP-1_42_ to immunogenicity.

**Figure 1:**
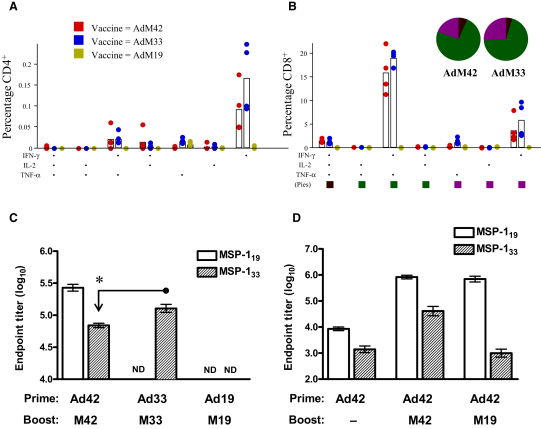
Immunogenicity of AdHu5-MVA-MSP-1 Recombinants (A and B) BALB/c mice were primed i.d. with AdHu5 expressing MSP-1_42_, MSP-1_33_, or MSP-1_19_ (Ad42, Ad33, or Ad19 respectively) and boosted i.d. 56 days later with the corresponding MVA vector (M42, M33, or M19). Fourteen days after boost, splenocytes were restimulated with MSP-1_33_ peptides, and the production of IFN-γ, TNF-α, and IL-2 by CD4+ (A) and CD8+ (B) T cells in the spleen was assessed using polychromatic flow cytometry. The functional compositions of the CD4^+^ and CD8^+^ T cell responses are shown, and these are grouped and color-coded according to the vaccine regime and number of cytokines secreted by each T cell population. The pie charts in (B) summarize the fractions of MSP-1_33_-specific CD8^+^ T cells that are positive for a given number of functions. Individual data points and mean percentage of the total response (open bars) are shown for each of the functional populations indicated on the x axis. No responses were detected above background following restimulation with MSP-1_19_ peptides (data not shown). Representative flow cytometry plots and the gating strategy are shown in Figure S1. (C and D) Total IgG serum antibody responses against MSP-1_19_ and MSP-1_33_ were measured by ELISA in mice primed and boosted as indicated. Mean responses ± SEM are shown (n = six mice per group). N.D. = not detected. ^∗^ p ≤ 0.05 in (C) (independent Student's t test comparing responses between the AdM42 and AdM33 groups). Similar results were obtained in three independent experiments.

Mice were immunized as described in Figure 1 using AdHu5-MVA vectors expressing the MSP-1_42_, MSP-1_33_, or MSP-1_19_ antigen (AdM42, AdM33, or AdM19, respectively), and antigen-specific immune responses were assayed. Relatively weak CD4^+^ T cell responses could be detected in the spleens of AdM42- and AdM33-immunized mice by intracellular cytokine staining (ICS) using a pool of overlapping peptides to MSP-1_33_ (Figure 1A). These CD4^+^ T cells almost exclusively produced only IFN-γ. In contrast, multifunctional CD8^+^ T cell responses to MSP-1_33_ were detected. These populations of T cells secreted various combinations of IFN-γ, TNF-α, and IL-2 and were of similar magnitude in mice immunized with AdM42 and AdM33 (Figure 1B). A clear hierarchy was also evident with a dominant population composed of IFN-γ^+^ TNF-α^+^ “double-positive” cells, followed by a population that produced only IFN-γ, and finally, a small population that was “triple-positive” and secreted all three cytokines, indicating only low-level production of IL-2 in comparison to IFN-γ and TNF-α. No regime induced MSP-1_19_-specific T cell responses that could be detected by ICS (data not shown). As previously reported, mice immunized with the AdM42 regime developed strong total IgG responses against the entire antigen, while in comparison, mice immunized with AdM33 developed a significantly higher total IgG response against MSP-1_33_ (Figure 1C). Interestingly, in contrast to mice immunized with AdM42, those immunized with AdM19 developed no detectable antibody response to MSP-1_19_ (Figure 1C). However, antibody responses in mice that were primed with Ad42 could be boosted by MVA expressing MSP-1_19_ (Ad42M19 regime; Figure 1D). These data indicate that essential T cell help for priming naive B cell responses against MSP-1_19_ comes from T cells induced against MSP-1_33_, but that there exists a different, and possibly reduced, requirement for CD4^+^ T cell help for memory B cells activated by a secondary exposure to the MSP-1_19_ antigen.

### Efficacy of AdHu5-MVA Recombinants against pRBC Challenge

**Figure 2:**
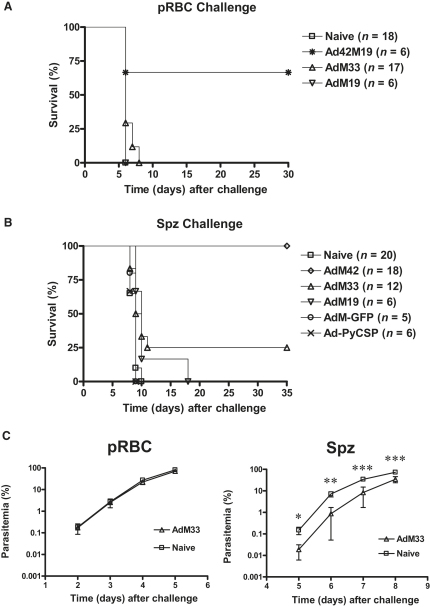
Survival against pRBC and Sporozoite Challenge (A–C) BALB/c mice were immunized as indicated. Fourteen days after the final immunization, these mice or naive controls were challenged i.v. with 10^4^ pRBCs (A) or 50 sporozoites (B), and Kaplan-Meier plots show survival over time. Data were pooled from multiple experiments, and the number of mice challenged in total (*n*) is indicated for each group. As shown in (C), mice immunized with AdM33 or naive controls were challenged with 10^4^ pRBCs or 50 sporozoites. Parasitemia in the blood was measured postchallenge by microscopy. Plots show the mean parasitemia ± SEM (n = six mice per group) for the first 4 days following patency. ^∗^ p ≤ 0.05, ^∗∗^ p ≤ 0.01, and ^∗∗∗^ p ≤ 0.001 (independent Student's t test comparing percent parasitemia at each time point between the immunized and naive control mice).

We have reported that AdM42 immunization can protect 76% of BALB/c mice (13 of 17) against a lethal challenge with 10^4^ *P. yoelii* pRBCs (Draper et al., 2008). That study did not confirm whether antibodies against MSP-1_33_ played a role in blood-stage immunity. Here, we assessed the protective efficacy of AdM33 and AdM19 immunization against a blood-stage challenge (Figure 2A). None of the mice immunized with AdM33 survived challenge, despite higher total IgG responses and similar T cell responses against MSP-1_33_ as compared to mice immunized with AdM42 (Figure 1). This confirms that no protection is mediated in this pRBC challenge model by MSP-1_33_-specific immune responses alone. Survival was seen in four of six mice (67%) immunized with Ad42M19, and notably, these mice possessed only high-titer IgG responses to MSP-1_19_ (Figure 1D). In contrast, none of the mice immunized with AdM19 was protected, which was unsurprising, given the absence of detectable immune responses induced by this vaccine regime (Figure 1). In agreement with our previous report, protection in this pRBC challenge model is dependent on MSP-1_19_-specific IgG.

### Efficacy of AdHu5-MVA Recombinants against Sporozoite Challenge

Malaria challenge with pRBCs does not mimic the natural mode of malaria infection by sporozoites. The efficacy of AdHu5-MVA-MSP-1 immunization against spz challenge was thus investigated (Figure 2B). After a lag period corresponding to the pre-erythrocytic infection, naive control mice succumbed to infection in an identical manner to those challenged with pRBCs and with near-identical parasitemia profiles (Figure S2A), suggesting that parasite virulence between the two challenge models is comparable. Nevertheless, all of the mice immunized with AdM42 developed patent blood-stage parasitemia (Figure S2B) but were able to control blood-stage parasite growth and clear the infection (Figure 2B). Indeed, following spz rather than pRBC challenge, blood-stage protection (using percent survival as the outcome measure) showed a significant increase, from 76% (Draper et al., 2008) to 100%, in mice immunized with AdM42, p = 0.046. Survival was also enhanced by a similar margin, to 25%, in mice immunized with AdM33 following spz challenge (Figures 2B and S2A), as compared to 0% in naive controls following spz challenge (p = 0.044) or 0% in mice immunized with AdM33 following pRBC challenge (Figure 2A). None of the mice immunized with AdM19 survived spz challenge.

We postulated that a number of factors could potentially contribute to an increase in vaccine efficacy following spz rather than pRBC challenge. These factors, in concert with MSP-1_19_-specific IgG, may enhance blood-stage protection mediated by AdM42 immunization, or in a small number of cases, lead to survival against blood-stage infection in mice immunized with AdM33 that lack protective antibodies. Indeed, we had consistently noted a significant reduction in blood-stage parasitemia in mice immunized with AdM33 on day 5 after spz challenge (∼48 hr after merozoite release from the liver), but not 48 hr after direct blood-stage infection by pRBC challenge (Figure 2C). We therefore sought to investigate a possible antiparasitic effect induced by AdM42 and AdM33 immunization against the liver-stage parasite.

### MSP-1_33_-Specific CD8^+^ T Cell Responses Mediate Partial Efficacy against the Liver-Stage Parasite

**Figure 3:**
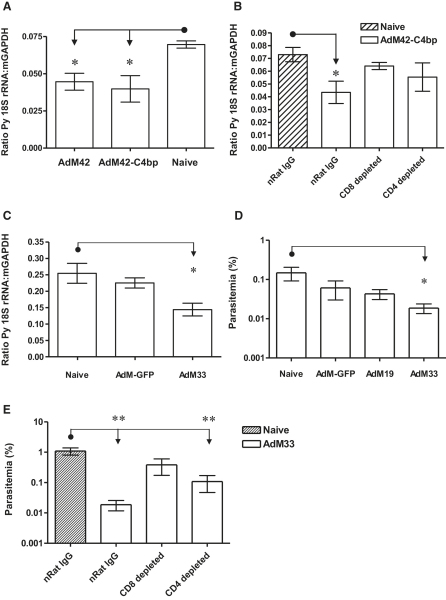
AdHu5-MVA-MSP-1 Immunization Efficacy against Liver-Stage Infection (A–E) BALB/c mice were immunized with AdM42 or AdM42-C4bp (A), AdM42-C4bp (B), or AdM33 or AdM-GFP vector controls (C), and challenged with 5000 sporozoites 14 days after the boost. Liver-stage parasite burden was quantified 48 hr postchallenge using an established real-time RT-PCR assay (Bruna-Romero et al., 2001). In (B), mice immunized with AdM42-C4bp were depleted of CD8^+^ or CD4^+^ T cells or control depleted with normal rat IgG (nRat IgG) prior to challenge. (D) shows blood-stage parasitemia on day 5 in BALB/c mice immunized as indicated or immunized with AdM33 and depleted of T cell subsets as indicated (E) and challenged with 50 sporozoites. The mean ratio or parasitemia ± SEM is shown (n = five or six mice per group). ^∗^ p ≤ 0.05 and ^∗∗^ p ≤ 0.01, comparing between all groups by one-way ANOVA.

The exoerythrocytic cycle of *P. yoelii* lasts approximately 50 hr (Killick-Kendrick and Peters, 1978). Given that AdM42 and AdM33 immunization elicits strong CD8^+^ T cell responses against MSP-1_33_ (Figure 1B), it is possible that these effector T cells can protect against the late exoerythrocytic schizonts that contain merozoites expressing MSP-1. To address this question, mice were immunized with AdM42 or with the previously described vaccines expressing MSP-1_42_ fused to mouse complement C4b-binding protein (C4bp) (Draper et al., 2008) and were challenged with 5000 sporozoites, and the liver-stage parasite burden was quantified using a real-time RT-PCR assay (Figure 3A). Mice immunized with both regimes showed a significant reduction in comparison to naive controls. These data are in agreement with challenge studies using 50 sporozoites, whereby the observed partial reduction in liver-stage parasite burden would not result in sterile immunity and hence not prevent blood-stage infection (Figures S2B and S2C). To confirm the role of T cells in mediating liver-stage immunity, mice were immunized with AdM42-C4bp and then depleted of T cells prior to spz challenge. Depletion of CD8^+^ T cells and, to a lesser extent, CD4^+^ T cells attenuated the vaccine-induced reduction in liver-stage parasite burden (Figure 3B).

Given that the CD8^+^ T cell response elicited by the AdM42 vaccine regime in BALB/c mice is targeted against epitopes within MSP-1_33_ (Sridhar et al., 2008), and given that the AdM33 regime described here induces a similar T cell response to AdM42 (Figure 1), we sought to confirm that this vaccine could mediate similar efficacy against the liver-stage forms of *P. yoelii*. BALB/c mice were immunized with AdM33 or vector controls (AdM-GFP). A significant reduction in liver-stage parasite burden was observed with AdM33 (Figure 3C), similar to that previously seen with AdM42. The reduced liver-stage burden with AdM33 was reflected in the levels of blood-stage parasitemia on day 5 following challenge with 50 sporozoites (Figure 3D). In vivo depletion of the CD8^+^ and CD4^+^ T cell subsets again confirmed that this protective effect is primarily dependent on CD8^+^ and not on CD4^+^ T cells, as noted by a loss of this reduction in blood-stage parasitemia on day 5 (Figure 3E). Partial efficacy against the liver-stage parasite in this model can thus be attributed to the T cell response against the MSP-1_33_ antigen when used alone or in the context of MSP-1_42_.

### Viral Vector Immunization Mediates Short-Term Efficacy against Sporozoite Challenge

During these spz challenge experiments (conducted 14 days after boosting), we noted a small and consistent but not statistically significant reduction in blood-stage parasitemia on day 5 postchallenge in mice immunized with AdM19 and AdM-GFP (Figure 3D). This effect was not apparent following challenge at a later time point (data not shown). It has been reported that potent Th1-type innate immune responses induced by CpG immunization can mediate short-term protection against *P. yoelii* spz challenge, which is dependent on IFN-γ and IL-12 and, in some cases, also NK cells, nitric oxide, and CD8^+^ T cells (Gramzinski et al., 2001). We tested the possibility that this may also be the case for viral vectored vaccines, whereby short-term protection can be mediated against spz challenge by vector control vaccines. Mice were primed with Ad-GFP and boosted with MVA-GFP 8 weeks later or simply primed with MVA-GFP alone. Mice were then challenged 2, 4, or 14 days later with 50 sporozoites. Eighty-three percent of the mice challenged on day 2 or day 4 were protected against challenge and did not develop blood-stage parasitemia (Table 1). This protective effect was the same following either a single immunization with MVA-GFP or AdM-GFP prime-boost, indicating a strong immune response against the MVA vector, rather than a strong boosting of the immune response against that of the GFP transgene, is accounting for protection (immune responses against GFP have been documented in BALB/c mice [Gambotto et al., 2000]). In vivo depletion studies suggested this protective effect is mediated by CD8^+^ and CD4^+^ T cells and NK cells, but not by macrophages (Table 1).

### AdM42 Immunization Protects Mice against a High-Dose Sporozoite Challenge

**Figure 4:**
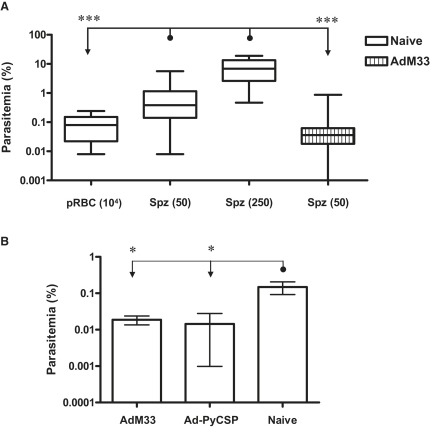
Blood-Stage Parasitemias in Naive and Immunized Mice 48 hr after Blood-Stage Infection (A) BALB/c mice immunized with AdM33 were challenged with 50 sporozoites (n = 18), or naive controls were challenged with 10^4^ pRBCs (n = 29), 50 sporozoites (n = 44), or 250 sporozoites (n = 8). Blood-stage parasitemia ∼48 hr post blood-stage infection was assessed by microscopy (day 5 post spz challenge and day 2 post pRBC challenge). Data were pooled from multiple experiments. Plots show median values, 25^th^–75^th^ percentiles, and range. One mouse in the AdM33 group had 0% parasitemia on day 5. (B) BALB/c mice were immunized with AdM33 or 1 × 10^8^ vp AdHu5-PyCSP and challenged with 50 sporozoites 14 days later. The mean parasitemia ± SEM is shown (n = five or six mice per group). ^∗^p ≤ 0.05 and ^∗∗∗^ p ≤ 0.001, comparing between all groups by one-way ANOVA.

Having established that AdM42 and AdM33 immunization can mediate an antiparasitic effect at the liver stage, we sought to assess whether the increased survival outcome against spz challenge was due to a reduction in the blood-stage inoculum from the liver. A single hepatic schizont of *P. yoelii* malaria will produce approximately 7500–8000 merozoites (Killick-Kendrick and Peters, 1978). Consequently, just two infected hepatocytes should release a larger blood-stage inoculum than the 10^4^ pRBCs used in the blood-stage challenge model. Levels of blood-stage parasitemia were compared 2 days post blood-stage infection in naive BALB/c mice challenged with 10^4^ pRBCs, 50 sporozoites (low dose), or 250 sporozoites (high dose) (Figure 4A). These data showed that spz challenge in naive mice leads to a significantly higher blood-stage parasitemia after 48 hr of blood-stage infection when compared to pRBC challenge. Mice immunized with AdM33 have a reduced liver-stage parasite burden (Figure 3C) but do not possess protective IgG. The same assessment of blood-stage parasitemia in mice immunized with AdM33 and challenged with 50 sporozoites showed, interestingly, that the mean parasitemia level was significantly reduced to one comparable with pRBC challenge (Figure 4A). These data indicate that if the amount of blood-stage inoculum from the liver was the sole determinant of survival outcome, then mice immunized with AdM33 and challenged with 10^4^ pRBCs or 50 sporozoites should show the same percent survival outcome, which was not the case (Figures 2A and 2B).

Vaccines developed against *P. yoelii* can show reduced efficacy against a high-dose challenge with 250 sporozoites (Sedegah et al., 2002). To confirm that protective efficacy was maintained at a higher blood-stage inoculum, mice were immunized with AdM42 and challenged with 250 sporozoites. All of the immunized mice (12 of 12) survived this higher dose challenge, compared to 0 of 8 naive control mice. Complete survival is thus maintained even after a high-dose *P. yoelii* spz challenge.

### An Effective T Cell Response against the Liver-Stage Parasite Does Not Lead to Defective Growth of Blood-Stage Parasites

An alternative explanation for these survival data could be defective blood-stage growth by parasites that were damaged by antigen- or vector-specific immune mechanisms in the liver. However, AdHu5 vaccines encoding the *P. yoelii* circumsporozoite protein (PyCSP) have been reported to induce highly protective CD8^+^ T cell responses against spz challenge (Rodrigues et al., 1997) but not to affect subsequent blood-stage survival. To confirm that parasites do not have defective blood-stage growth following an effective liver-stage immune response, mice were immunized with AdM33 or given a single immunization of 1 × 10^8^ viral particles (vp) AdHu5-PyCSP and then challenged with 50 sporozoites. This dose of AdHu5-PyCSP vaccine was titered to give a similar reduction in liver-stage parasite burden as AdM33 but not sterilizing immunity. Mean blood-stage parasitemias, as measured on day 5 postinfection, were comparable between the immunized groups, and both were significantly lower than naive controls (Figure 4B). However, in the case of the AdHu5-PyCSP vaccine, none of the mice survived blood-stage infection (Figure 2B). These data indicate that *P. yoelii* parasites released into the blood are viable despite a partially effective response at the liver-stage.

### Priming a CD4^+^ T Cell Response against MSP-1_33_ Leads to the Faster Induction of De Novo Antibody Responses against MSP-1_19_

We hypothesized that an alternative explanation for the observed increase in survival may be due to an enhanced de novo production of protective anti-MSP-1_19_ IgG responses in mice previously immunized with AdM33. In this scenario, CD4^+^ T cell helper responses would be primed and ready to assist naive B cells immediately upon natural exposure to MSP-1_19_ antigen. Serum antibody responses were measured in all mice prechallenge and ∼120 hr post blood-stage infection. Indeed, following spz challenge, 7 of 12 of the mice immunized with AdM33 had developed detectable IgG responses against MSP-1_19_ within 5 days (Figure S3). Only two other mice within all of the other vaccinated or control groups possessed a detectable IgG antibody response against this domain. However, the levels of these detected antibodies were far below the protective threshold established in previous work (Draper et al., 2008), and furthermore, a similar number (5 of 11) of mice immunized with AdM33 and challenged with pRBCs also developed detectable MSP-1_19_ IgG responses, but did not survive. So these weak antibody responses by themselves are highly unlikely to account for protective efficacy at this time point.

### Viral-Vector Immunization Enhances the IFN-γ Serum Cytokine Response against Blood-Stage Parasites Following Sporozoite but Not pRBC Challenge

**Figure 5:**
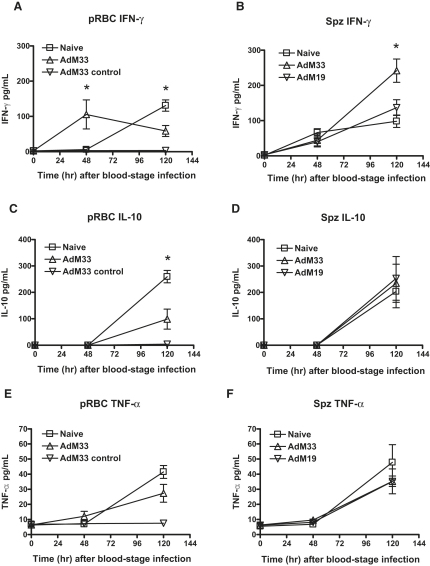
Serum Cytokine Responses Following pRBC and Sporozoite Challenge (A–F) BALB/c mice were immunized as indicated and challenged with 10^4^ pRBCs (A, C, and E) or 50 sporozoites (B, D, and F) or not challenged (“AdM33 control”) (A, C, and E). Serum was collected prechallenge (0 hr), 48 hr post blood-stage infection (day 5 post spz challenge and day 2 post pRBC challenge), and 120 hr post blood-stage infection (day 8 post spz challenge and day 5 post pRBC challenge). Serum cytokine levels were assayed by flow cytometry using a cytometric bead array assay. Mean levels ± SEM are shown (n = five or six mice per group) for IFN-γ (A and B), IL-10 (C and D), and TNF-α (E and F). ^∗^ p ≤ 0.05, comparing between groups (independent Student's t test in [A] and [C]), and one-way ANOVA in (B). Similar results were obtained in two independent experiments.

A balanced cytokine response is reported to be essential for the control of blood-stage parasite growth. The ratio of protective proinflammatory cytokines (such as IFN-γ and TNF-α) to regulatory cytokines (such as IL-10 and TGF-β), has been shown to determine the outcome of natural blood-stage infection with lethal versus nonlethal *P. yoelii* (Omer et al., 2003) and to correlate with the growth rate of blood-stage parasites in malaria-naive human volunteers (Walther et al., 2006). We hypothesized that exposure of vaccine-induced T cells to MSP-1 at the liver stage (after spz challenge) might allow for the development of a qualitatively or quantitatively different and more protective cytokine response than is developed in mice challenged only with pRBCs, who will not have had this antecedent activation of T cells prior to blood-stage infection. Mice were thus immunized and then challenged with pRBCs or sporozoites. Blood samples were taken prechallenge and 2 days and 5 days after the onset of blood-stage infection, and serum cytokines were assessed using a cytometric bead array assay. Mice that were immunized with AdM33 and not challenged (“AdM33 control”) showed no detectable serum cytokine responses at any time point (Figures 5A, 5C, and 5E). Naive mice challenged with pRBCs or sporozoites showed a similar induction of IFN-γ, TNF-α, and IL-10 in the serum by 120 hr post blood-stage infection (Figures 5A–5F). Yet mice that were immunized with AdM33 and then challenged with pRBCs showed a significant early induction of IFN-γ after 48 hr, which was then significantly downregulated by 120 hr, when compared to naive controls (Figure 5A). The level of IL-10 in the serum mirrored this trend at the 120 hr time point in immunized mice and was significantly lower than in the naive controls (Figure 5C). However, this was not the case in mice immunized with AdM33 and challenged with sporozoites. Their mean serum levels of IFN-γ and IL-10 were similar to naive controls after 48 hr (Figures 5B and 5D), and IFN-γ was not downregulated by 120 hr (as it was for pRBC-challenged mice) but was in fact significantly higher (Figure 5B). In the case of mice immunized with AdM19, AdM-GFP, or Ad-PyCSP, a similar trend was maintained, but none of these groups possessed significantly higher levels of IFN-γ in the serum as compared to naive controls at this time point (Figures 5B and S4). Serum levels of TNF-α were similar in all the challenge groups (Figures 5E and 5F). These data indicate that the increased survival in mice immunized with AdM33 and then challenged with spz rather than pRBCs is associated with a more effective and maintained induction of IFN-γ—a pattern similar to that seen in the nonlethal 17XNL strain of *P. yoelii* and contrasting with the lethal 17XL parasite strain (Omer et al., 2003).

## Discussion

We report a vaccination strategy that can induce protective multistage immunity against malaria by using viral vectors that are suitable for human use (Catanzaro et al., 2006; Hill, 2006). These viral vaccines expressing MSP-1_42_, like protein vaccines, allow for the induction of highly protective IgG against MSP-1_19_, which accounts for the major protective mechanism in this model against the blood-stage parasite. However, unlike protein vaccines, the concomitant induction of MSP-1_33_-specific T cells and, to a lesser extent, antigen-nonspecific vector-induced responses can provide partial liver-stage efficacy in this mouse model and should allow for combination with similar partially effective pre-erythrocytic antigen vectors (Hill, 2006). Following spz challenge of immunized mice, the significantly reduced blood-stage inoculum is associated with a maintained induction of IFN-γ in the blood. This in turn correlates with increased survival in mice immunized against MSP-1_33_ or complete survival in mice immunized with MSP-1_42_ (and which also possess protective IgG against MSP-1_19_). These data have important implications for the choice of clinical challenge model used to assess new candidate blood-stage malaria vaccines and demonstrate that the induction of effective immune responses against both the liver and blood stages of malaria infection can lead to increased vaccine efficacy.

The need for CD4^+^ T helper cell responses against MSP-1_33_ to prime antibody responses against MSP-1_19_ is in agreement with studies that show MSP-1_19_ is refractory to antigen processing (Hensmann et al., 2004). Similarly, following immunization of naive humans with an MSP-1_42_ formulation in a phase I clinical trial, T cell responses were identified against MSP-1_33_ and not MSP-1_19_ (Huaman et al., 2008). The ability of the vectors expressing MSP-1_19_ to boost but not prime these antibody responses would support the use of MSP-1_19_ recombinant protein vaccines to boost antibody responses primed by viral vectors expressing MSP-1_42_.

CD8^+^ T cell responses were detected only against MSP-1_33_, and their ability to mediate liver-stage protection is consistent with the expression of MSP-1 during liver-stage infection (Sacci et al., 2005) and a previous MSP-1 study that demonstrated comparable liver-stage efficacy (Kawabata et al., 2002) using a T cell-inducing vaccine technology that required intravenous vaccine administration. The inability of MSP-1_33_-specific T cells and IgG to protect against a pRBC challenge is in agreement with some (Ahlborg et al., 2002) but not all (Wipasa et al., 2002) previous studies. Taken together, these data argue for the use of MSP-1_42_- rather than MSP-1_19_-based vaccines to increase the likelihood of inducing multistage protection.

Antimalarial factors that can reduce liver-stage burden, including bednets (Lengeler, 2004), immunogenetic risk factors (Hill et al., 1991), and the RTS,S/AS02 vaccine (Alonso et al., 2004), can ameliorate the severity of subsequent blood-stage infection in naturally exposed individuals. However, this effect has not been reported in the literature for preclinical studies of pre-erythrocytic-stage vaccine candidates. In our report, enhanced survival following spz challenge appears to be due to vaccine-induced effector mechanisms acting against both the liver and blood stages of infection. Immunization with AdM42 or AdM33 leads to a reduced merozoite load emerging from the liver, which is then associated with a more effective induction of protective IFN-γ early after blood-stage infection. This is in comparison to pRBC challenge where this cytokine appears to be induced faster but then downregulated. Although blood-stage parasites have been reported to modulate immune cell function (Urban et al., 1999; Wipasa et al., 2001; Wykes et al., 2005), why cytokine induction should be different following inoculation of the blood via sporozoites or pRBCs remains unknown.

In one study using *P. chabaudi* parasites, differences in the cytokine responses of CD4^+^ T cells were noted after the infection of naive mice with pRBCs versus mosquito bite (Fonseca et al., 2007). In this model, Th1 cytokine responses were in fact reduced following infection by the route of natural transmission. In our model, however, antigen-specific responses have been primed by vaccination, and so temporal activation of MSP-1-specific T cell populations, innate effector cell types, or antigen-presenting cells in the liver prior to blood-stage infection may be an important factor in determining outcome. It should be noted that this effect occurred significantly only in mice immunized with AdM33, and not those immunized with Ad-PyCSP. The activation of antigen-specific T cells during liver infection may only influence the blood-stage infection if antigen expression is maintained by the parasite in the blood stage, explaining why significantly increased serum levels of IFN-γ were found in mice immunized against MSP-1 but not CSP. Our data did not indicate that, following a partially effective T cell response in the liver, the remaining parasites were damaged or less virulent in the blood stage. Similarly, an earlier induction of protective antibody responses against MSP-1_19_ did occur in mice possessing CD4^+^ T cells primed against MSP-1_33_ before challenge; however, these were not universal and could not alone count for the differences in survival observed between the two challenge models.

The assessment of blood-stage malaria vaccine efficacy in phase I/IIa clinical trials can use one of two models. The spz clinical challenge model allows for an assessment of complete or partial protection at the liver stage in immunized volunteers, and although blood-film positivity requires drug cure, partial blood-stage efficacy can be observed prior to this in partially protected volunteers by using real-time PCR (Thompson et al., 2008). An alternative blood-stage parasite clinical challenge model uses a smaller blood-stage inoculum (compared to spz challenge), which leads to a longer prepatent period and more time for assessment of blood-stage parasite growth rates by real-time PCR (Sanderson et al., 2008) but to date has only been utilized in three clinical studies (Lawrence et al., 2000; Pombo et al., 2002; Sanderson et al., 2008). With many promising new blood-stage vaccine candidates entering the clinical phase, careful consideration needs to be given to the choice of challenge model employed to assess efficacy prior to field studies, including which effector mechanisms may contribute to protective immunity and whether these may also be effective against the liver stage.

The concept of inducing multistage protection has been a holy grail of malaria vaccine design, and many attempts to do this have been reported. Here, we report that a single antigen can induce significant protective efficacy at the pre-erythrocytic and erythrocytic stages of infection and following only two immunizations. This reflects, in part, the unusual ability of this vectored vaccine regime to induce both high titers of antibody and strong T cell responses. These data encourage the development of vaccine technologies that can induce strong antibody and T cell responses against multistage malaria antigens in order to achieve maximal protective efficacy.

## Experimental Procedures

### Generation of Recombinant MVA and AdHu5 Vaccines

The generation of AdHu5 and MVA vectors expressing *P. yoelii* YM MSP-1_42_ and MSP-1_42_-C4bp has been described (Draper et al., 2008). Those expressing *P. yoelii* YM MSP-1_33_, MSP-1_19_, and *P. yoelii* 17XL CSP were made in an identical manner. Specific cloning details can be found in the Supplemental Data. MSP-1_33_, MSP-1_19_, and CSP were expanded by PCR from template DNA and cloned into the MVA shuttle vector pMVA.GFP, and recombinant MVAs were generated as previously described. Recombinant AdHu5 vaccines were constructed using the ViraPower Adenoviral Expression System (Invitrogen) and purified using the Adenopure Kit (PureSyn; Malvern, PA). AdHu5 and MVA vectors, expressing a synthetic construct consisting of GFP fused at the N terminus to one HLA class I- and one class II-restricted epitope from influenza virus, were generated in the same manner and used as vector controls for immunization.

### Animals and Immunizations

Female BALB/c (H-2^d^) mice (Harlan, UK), 6–8 weeks old, were used in all experiments. All procedures were carried out under the terms of the UK Animals (Scientific Procedures) Act Home Office Project License and were approved by the University of Oxford Animal Care and Ethical Review Committee. Mice were immunized intradermally (i.d.) with 5 × 10^7^ pfu MVA vaccines or 5 × 10^10^ vp AdHu5 vaccines diluted in PBS and administered bilaterally into the ears.

### ELISA

Serum was analyzed for antibodies as previously described (Draper et al., 2008). Recombinant GST fusion proteins (GST-MSP-1_33_ or GST-MSP-1_19_) or GST control were adsorbed to 96-well Nunc-Immuno Maxisorp plates at a concentration of 2 μg/mL in PBS. Briefly, plates were washed with PBS containing 0.05% Tween 20 (PBS/T) and blocked with 10% skimmed milk powder in PBS/T. Sera were diluted to 1:100, added in duplicate wells, and serially diluted. Bound antibodies were detected using alkaline phosphatase-conjugated goat anti-mouse total IgG. Plates were developed by adding *p*-nitrophenylphosphate substrate. Optical density was read at 405 nm (OD_405_). Endpoint titers were taken as the x axis intercept of the dilution curve at an absorbance value 3× standard deviations greater than the OD_405_ for naive mouse serum (typical cut-off OD_405_ for positive sera = 0.15). A high-titer serum sample was included in all assays as a reference control. All GST control ELISAs were negative (data not shown).

### Multiparameter Flow Cytometry

Cytokine secretion by mouse splenocytes was assayed as described previously (Draper et al., 2008). Briefly, splenocytes were restimulated in the presence of GolgiPlug (BD Biosciences) for 5 hr at 37°C with pools of 15 mer peptides overlapping by 10 amino acids (final concentration: 5 μg/mL for each peptide). Overlapping peptide pools corresponded to MSP-1_33_ containing 52 peptides or MSP-1_19_ containing 19 peptides. Staining antibodies were specific for mouse and purchased from eBioscience. After blocking Fc receptors with anti-CD16/CD32, cells were surface stained for 30 min at 4°C with Pacific Blue-labeled anti-CD8α and APC-Alexa Fluor 750-labeled anti-CD4. Cells were permeabilized in Cytofix/Cytoperm solution as per manufacturer's instructions (BD Biosciences). Intracellular cytokines were stained with PE-labeled anti-IFN-γ, APC-labeled anti-TNF-α, and FITC-labeled anti-IL-2. Samples were analyzed using a CyAn ADP Flow Cytometer (Dako; Ely, UK) and FlowJo version 8.7 (Tree Star, Inc.; USA). The Boolean gate platform was used with individual cytokine gates to create all possible response combinations. Pestle 1.5 and SPICE 4.1 software programs (Mario Roederer; VRC, NIAID, NIH) were used to analyze the T cell response profiles. Background responses in unstimulated control cells were subtracted from the stimulated response.

### In Vivo Depletions

In vivo-depleting monoclonal antibodies (mAbs) were purified by protein G affinity chromatography from hybridoma culture supernatants. Anti-CD4 GK1.5 (rat IgG2a) and anti-CD8 2.43 (rat IgG2a) were diluted in sterile PBS. Normal rat IgG (nRatIgG) was purchased from Sigma and purified by the same method. For depletion of CD4^+^ or CD8^+^ T cells, mice were injected intraperitoneally (i.p.) with 200 μg of the relevant mAb on days −2 and −1 before and on the day of challenge. In vivo T cell depletion was confirmed by flow cytometry of surface-stained peripheral blood mononuclear cells (PBMCs) from depleted and control mice. To inhibit macrophages, mice were administered 1 mg of Carrageenan (CGN) (Sigma) i.p. on days 0, 1, and 2 relative to challenge on day 0. To deplete NK cells, mice received a single dose of 200 μl of anti-asialo GM1 antiserum (Alpha Labs, UK) diluted 1:8 in 0.5× PBS on days −2, 0, and +2 relative to challenge.

### *P. yoelii* (Strain YM) Challenge

Mice were challenged with 10^4^ pRBCs by the intravenous (i.v.) route as previously described (Draper et al., 2008). Blood-stage parasitemia was monitored from day 2 postchallenge by microscopic examination of Giemsa-stained blood smears. Levels of parasitemia are calculated as the percentage of pRBCs. Mice were deemed uninfected in the absence of patent parasitemia in 50 fields of view. Infection was considered lethal when parasitemia exceeded 80%, at which point animals were euthanized. For spz challenge, salivary glands of infected *Anopheles stephensi* mosquitoes were dissected and homogenized in RPMI 1640 medium to release parasites. Mice were challenged i.v. with 50 or 250 sporozoites, and parasitemia was monitored from day 5.

### Quantification of *P. yoelii* Parasite Burden in the Liver

Specific details can be found in the Supplemental Data. Mice were challenged with 5000 sporozoites. Livers were harvested after 48 hr and snap frozen in liquid nitrogen. Whole livers were homogenized in Trizol (Invitrogen), and total liver RNA was extracted using chloroform. RNA was digested with RNase-Free DNase (QIAGEN, UK) and purified. Two micrograms of RNA was reverse transcribed to cDNA using Omniscript (QIAGEN). cDNA encoding *P. yoelii* 18S rRNA or mouse glyceraldehyde-3-phosphate dehydrogenase (mGAPDH) was amplified in triplicate by quantitative real-time PCR using a Rotor-Gene 3000 (Corbett Life Science, Australia). Twenty picomoles of specific primer pairs were included in the QuantiTect RT-PCR Buffer (QIAGEN) containing the dsDNA-specific fluorescent dye SYBR Green I. The temperature profile of the reaction was previously described (Bruna-Romero et al., 2001). The threshold cycle value (C_T_) of each PCR was converted to a DNA copy number equivalent by reading against standard curves generated by amplifying 10-fold dilutions of plasmid containing the relevant target cDNA molecule. The liver-stage parasite burden was determined for each sample as the ratio of the DNA copy number equivalent measured for the *P. yoelii* 18S rRNA over the DNA equivalent for mGAPDH.

### Serum Cytokine Analysis

A Cytometric Bead Array (Mouse Inflammation Kit, CBA; BD Biosciences) was used to study cytokine levels in mouse serum. Serum was collected from tail vein bleeds, stored on ice, centrifuged, and frozen prior to analysis. Serum was diluted 1:2 and analyzed according to manufacturer's instructions.

### Statistical Analysis

Data were analyzed using GraphPad Prism version 4.03 for Windows (GraphPad Software; San Diego, CA). Data set normality was assessed by Kolmogorov-Smirnov test. ELISA endpoint titers were normalized by log_10_ transformation. Independent-samples Student's t test was performed to compare mean responses between two groups. One-way between-groups ANOVA with post hoc Bonferroni or Dunnett's correction, as appropriate, was used to compare responses between more than two groups. Survival analysis was performed using Fisher's exact test (two-tailed). A value of p ≤ 0.05 was considered significant in all cases.

## Figures and Tables

**Table 1:** Vector-Mediated Protective Immunity Against Sporozoite Challenge

Immunization Regime	Day Challenged	Treatment	No. Infected / Total	% Protection	P value
AdM-GFP	+2	N/A	1/6	83%	0.015
AdM-GFP	+4	N/A	1/6	83%	0.015
M-GFP	+4	N/A	1/6	83%	0.015
AdM-GFP	+14	N/A	6/6	0%	
Naive	N/A	N/A	6/6	0%	

AdM-GFP	+2	Control IgG	1/6	83%	
AdM-GFP	+2	Anti-CD8^+^ T cells	3/4	25%	0.19
AdM-GFP	+2	Anti-CD4^+^ T cells	3/4	25%	0.19
AdM-GFP	+2	Anti-Macrophages	1/4	75%	
AdM-GFP	+2	Anti-NK cells	3/4	25%	0.19
Naive	N/A	Control IgG	4/4	0%	
Naive	N/A	Anti-Macrophages	4/4	0%	

Mice were immunized with AdHu5-MVA prime-boost or MVA alone expressing GFP and challenged with 50 sporozoites 2, 4, or 14 days after the MVA immunization. Mice were depleted as described in Experimental Procedures. *P* values were calculated using Fisher's exact test (two-tailed) by comparing protection in immunized mice versus naive controls or protection in mice receiving treatment versus controls.

## Supplemental Data

Document S1. Supplemental Experimental Procedures, Supplemental References, and Four Figures
