# Supplementary material for: Recombinant Viral Vaccines Expressing Merozoite Surface Protein-1 Induce Antibody- and T Cell-Mediated Multistage Protection against Malaria
Source: Cell Host Microbe. 2009 Jan 22;5(1):95–105. doi: 10.1016/j.chom.2008.12.004 (PMC2663714; doi:10.1016/j.chom.2008.12.004)

Supplemental Data

Cell Host & Microbe, *Volume 5*

Recombinant Viral Vaccines Expressing Merozoite Surface Protein-1 Induce Antibody- and T Cell-Mediated Multistage Protection against Malaria

Simon J. Draper, Anna L. Goodman, Sumi Biswas, Emily K. Forbes, Anne C. Moore, Sarah C. Gilbert, and Adrian V.S. Hill

Supplemental Experimental Procedures:

Generation of Recombinant MVA and AdHu5 Vaccines.

The generation of AdHu5 and MVA vectors expressing *P. yoelii* YM MSP-1_{42} and MSP-1_{42}-C4bp has been described previously (Draper et al., 2008). Those expressing *P. yoelii* YM MSP-1_{33}, MSP-1_{19} and *P. yoelii* 17XL CSP were made in an identical manner except as follows. MSP-1_{33} (amino acids (aa) 1394-1657) was amplified by PCR using the 42F forward primer 5'-GTC GAC TCC GAA GAT GCA CCA GAA AAA GAT AT-3' and the 33R reverse primer 5'-GCA TGC GGA TCC TCA GTC TAG TCC TAG CAA AGG GTT GGG AAT TAC ACC TAA TAA ATC CAT ACC ATC CAT GTT-3' from plasmid λPyM4.3 (Lewis, 1989). MSP-1_{19} (amino acids (aa) 1649-1757) was amplified using the 19F forward primer 5'-GGA TCC GTC GAC ATG GAT GGT ATG GAT TTA TTA GGT G-3' and the 42R reverse primer 5'-GCA TGC GGA TCC TCA GTC TAG ACC TAG CAA AGG GTT AGG AAT TCC CAT AAA GCT GGA AGA ACT ACA GAA TAC-3'. *P. yoelii* CSP (aa 1-356) was expanded by Phusion PCR (New England Biolabs, Ipswich UK) using the PyCSPF forward primer 5'-GTC GAC ATG AAG AAG TGT ACC ATT TTA GTT GTA GCG-3' and PyCSPR reverse primer 5'-GCA TGC GGA TCC TCA GTC TAG ACC TAG CAA AGG GTT AGG AAT TCC TAA TGA ATT GCT TAC AAT ATT AAA TAT ACT TGA-3' from template DNA (Rodrigues et al., 1997) (a kind gift from M. Tsuji, New York University, USA). Constructs were

cloned into the MVA shuttle vector, pMVA.GFP and recombinant MVAs generated as previously described (Draper et al., 2008). Recombinant AdHu5 vaccines were constructed as previously described (Draper et al., 2008) using the ViraPower Adenoviral expression system (Invitrogen), and purified using the Adenopure Kit (PureSyn, Malvern, Pennsylvania, USA). AdHu5 and MVA vectors, expressing a synthetic construct consisting of green fluorescent protein (GFP) fused at the N-terminus to the HLA class I-restricted epitope GILGFVFTL (Gotch et al., 1987) and HLA class II-restricted epitope FVFTLTVPS from influenza virus matrix protein (Linnemann et al., 2000), were generated in the same manner and used as controls for vector immunization.

Quantification of *P. yoelii* Parasite Burden in the Liver

Mice were challenged as above with 5,000 sporozoites. Livers were harvested after 48 hours and snap frozen in liquid nitrogen. Whole livers were homogenized in Trizol (Invitrogen) using a Dispomix (Thistle Scientific, Glasgow, UK). Total liver RNA was extracted using chloroform as previously described (Briones et al., 1996) and quantified using a nanodrop. 40μg RNA was digested with RNase-free DNase (Qiagen, Crawley, West Sussex, UK) and purified using RNeasy MinElute Cleanup Kit (Qiagen). 2μg RNA was reverse transcribed to cDNA using Omniscript (Qiagen), random hexamer primers (Promega, Southampton, UK), oligo-dT, and RNasin Plus inhibitor (Promega). cDNA encoding *P. yoelii* 18S rRNA or mouse glyceraldehyde-3-phosphate dehydrogenase (mGAPDH) were amplified in triplicate by quantitative real-time PCR using a Rotor-Gene 3000 (Corbett Life Science, Sydney, Australia). Twenty pmoles of either specific primer pair (PyF 5'-GGG GAT TGG TTT TGA CGT TTT TGC G-3' and PyR 5'-AAG

CAT TAA ATA AAG CGA ATA CAT CCT TAT-3’; mGF 5’-TTC ACC ACC ATG GAG AAG GC-3’ and mGR 5’-GGC ATG GAC TGT GGT CAT GA-3’) were included in the QuantiTect RT-PCR buffer (Qiagen) containing the dsDNA-specific fluorescent dye SYBR Green I. The temperature profile of the reaction was previously described (Bruna-Romero et al., 2001). The threshold cycle value (C_T) of each PCR was converted to a DNA copy number equivalent by reading against standard curves generated by amplifying 10-fold dilutions of plasmid containing the relevant target cDNA molecule. The liver-stage parasite burden was determined for each sample as the ratio of the DNA copy number equivalent measured for the *P. yoelii* 18S rRNA over the DNA equivalent for mGAPDH.

Supplemental References:

Briones, M. R., Tsuji, M., and Nussenzweig, V. (1996). The large difference in infectivity for mice of Plasmodium berghei and Plasmodium yoelii sporozoites cannot be correlated with their ability to enter into hepatocytes. Mol Biochem Parasitol *77*, 7-17.

Bruna-Romero, O., Hafalla, J. C., Gonzalez-Aseguinolaza, G., Sano, G., Tsuji, M., and Zavala, F. (2001). Detection of malaria liver-stages in mice infected through the bite of a single Anopheles mosquito using a highly sensitive real-time PCR. Int J Parasitol *31*, 1499-1502.

Draper, S. J., Moore, A. C., Goodman, A. L., Long, C. A., Holder, A. A., Gilbert, S. C., Hill, F., and Hill, A. V. (2008). Effective induction of high-titer antibodies by viral vector vaccines. Nat Med *14*, 819-21.

Gotch, F., Rothbard, J., Howland, K., Townsend, A., and McMichael, A. (1987). Cytotoxic T lymphocytes recognize a fragment of influenza virus matrix protein in association with HLA-A2. Nature *326*, 881-882.

Lamoreaux, L., Roederer, M., and Koup, R. (2006). Intracellular cytokine optimization and standard operating procedure. Nat Protoc *1*, 1507-1516.

Lewis, A. P. (1989). Cloning and analysis of the gene encoding the 230-kilodalton merozoite surface antigen of Plasmodium yoelii. Mol Biochem Parasitol *36*, 271-282.

Linnemann, T., Jung, G., and Walden, P. (2000). Detection and quantification of CD4(+) T cells with specificity for a new major histocompatibility complex class II-restricted influenza A virus matrix protein epitope in peripheral blood of influenza patients. J Virol *74*, 8740-8743.

Rodrigues, E. G., Zavala, F., Eichinger, D., Wilson, J. M., and Tsuji, M. (1997). Single immunizing dose of recombinant adenovirus efficiently induces CD8+ T cell-mediated protective immunity against malaria. J Immunol *158*, 1268-1274.

Supplemental Figure 1. Gating strategy for analysis of MSP-1-specific T cell responses in the spleen. Representative flow cytometry plots are shown for the analysis of $CD8^+$ T cell responses from a mouse immunized with AdM33. Initial gating used (from top left to top right) forward scatter area (FSC-A) *versus* forward scatter linear (FSC-L) to remove doublet events and select singlet cells; a FSC-A *versus* side scatter area (SSC-A) gate to capture small lymphocytes; then following this, events were gated through CD4 *versus* IFN-γ to select the total CD4 negative ($CD4^-$) cell population, and finally CD8 *versus* IFN-γ to select the total CD8 positive ($CD8^+$) cell population (Lamoreaux et al., 2006). Percentages refer to the % of cells in the current gate as a fraction of the preceding gated population, for example in the plots shown 70.2% of lymphocytes are $CD4^-$, and of this $CD4^-$ population, 23.0% are $CD8^+$. The opposite strategy (gating for $CD8^-$ and then $CD4^+$ cells) was used to analyze $CD4^+$ T cell responses. Subsequently (lower panels) IL-2, TNF-α and IFN-γ cytokine responses for the $CD4^-$ $CD8^+$ T cell population were analyzed using bivariate plots (Lamoreaux et al., 2006). Percentages refer to the % of $CD4^-$ $CD8^+$ cells that express the specific cytokine. Responses for each cytokine are shown following re-stimulation with MSP-1_{33} peptides (top row), MSP-1_{19} peptides (middle row) and unstimulated control (bottom row). In the case of AdM33 immunization, the MSP-1_{19} peptides acted as an internal peptide control, and vice versa for the MSP-1_{33} peptides in the case of AdM19 immunization. Background responses in unstimulated control cells were subtracted from the stimulated response during the analysis.

Supplemental Figure 2. Protection against *P. yoelii* challenge. (A) BALB/c mice immunized with AdM33 or naïve controls were challenged with 10^4 pRBCs or 50 spz. Parasitemia in the blood was measured post-challenge by microscopy. A representative result is shown (*n* = 6 mice per group) for up to one month post-challenge. † indicates the point at which mice were euthanized. Other groups of mice were immunized with **(B)** AdM42 or **(C)** AdM42-C4bp and were challenged with 50 spz 14 d after boost (*n* = 6 mice per group). Percentage survival, including all repeat experiments, is indicated in square parentheses. All of the naïve unimmunized control mice succumbed to *P. yoelii* blood-stage infection within six days, as shown in **(A)**. Similar results were obtained in two or three independent experiments.

Supplemental Figure 3. Induction of *de novo* antibody responses against MSP-1_{19} post-challenge. Mice were immunized and challenged as indicated. Total IgG serum antibody responses against MSP-1_{19} were measured by ELISA 120 h post blood-stage infection (day 8 post sporozoite challenge and day 5 post pRBC challenge). All samples were negative pre-challenge (data not shown). Numbers of mice with detectable responses are indicated.

Supplemental Figure 4. Serum cytokine responses following spz challenge. BALB/c mice were immunized as indicated and challenged with 50 spz. Serum was collected 120 h post blood-stage infection (day 8 post sporozoite challenge). Serum cytokine levels were assayed by flow cytometry using a cytometric bead array assay. Mean levels ±

s.e.m. are shown for IFN-γ (n = 5-6 mice per group). * $P \leq 0.05$ comparing between groups by one-way ANOVA.

Supplemental Figure 1

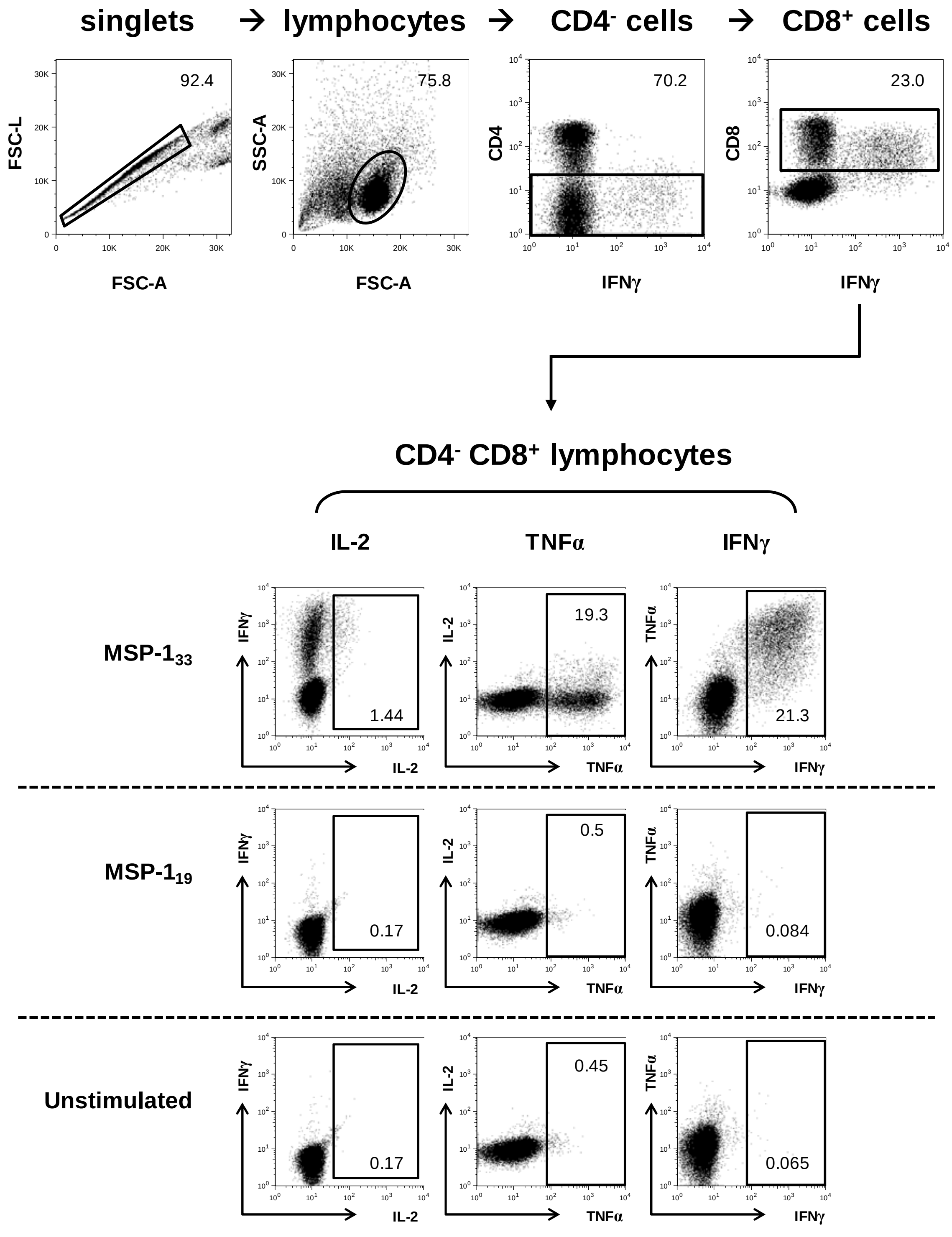

Supplemental Figure 2

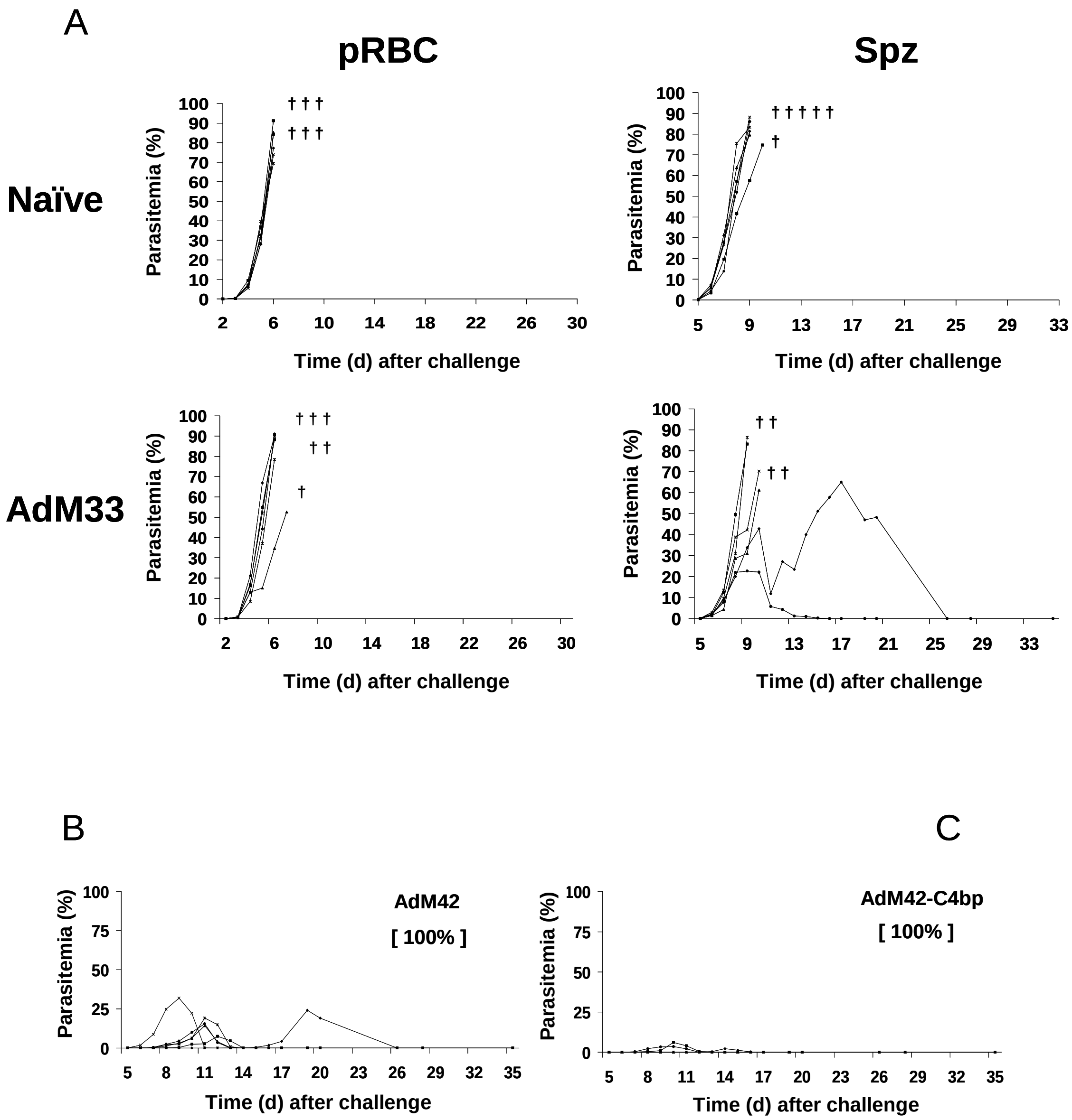

Supplemental Figure 3

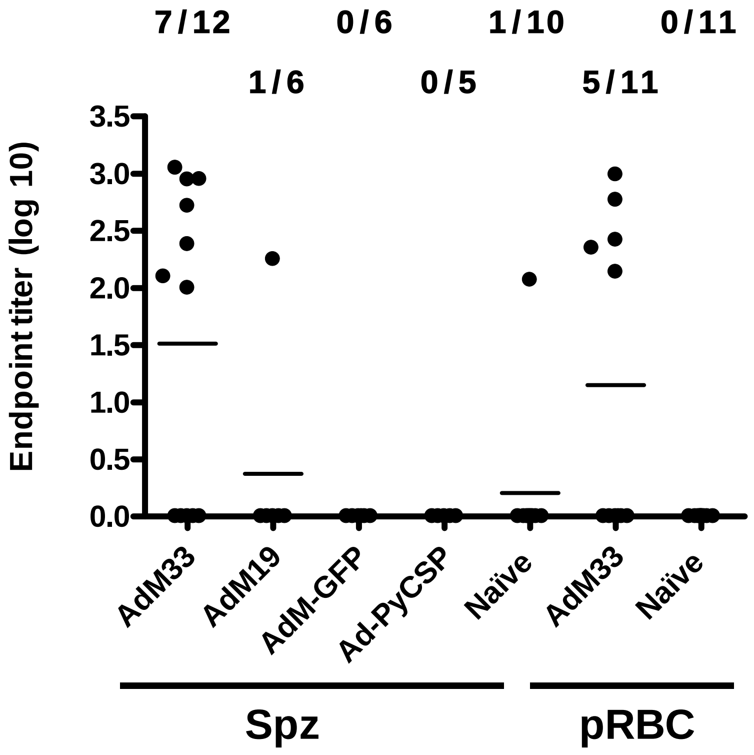

Supplemental Figure 4

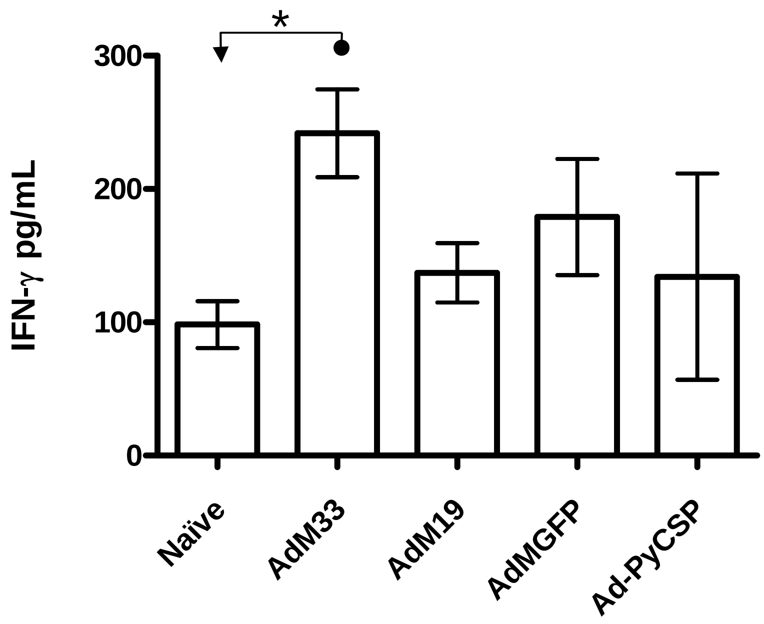

Supplement: Document S1. Supplemental Experimental Procedures, Supplemental References, and Four Figures [file mmc1.pdf]
